# Issue Information

**DOI:** 10.1002/lrh2.10195

**Published:** 2020-01-20

## Abstract

No abstract is available for this article.

